# The impact of student psychological empowerment on class stickiness

**DOI:** 10.3389/fpsyg.2025.1615370

**Published:** 2025-11-10

**Authors:** Hongping Fei, Junhui Zhang, Weilin Xiang, Haifeng Qi

**Affiliations:** School of Business, East China University of Science and Technology, Shanghai, China

**Keywords:** student engagement, psychological empowerment, psychological ownership, class stickiness, customer engagement

## Abstract

**Introduction:**

Sustaining student engagement (“class stickiness”) in hybrid/online courses is essential for learning and retention. Grounded in customer-engagement theory, we test a sequential model where student psychological empowerment fosters psychological ownership and involvement, which in turn drive class stickiness and course evaluation.

**Methods:**

A cross-sectional survey was administered to 320 Chinese undergraduates in blended courses. Validated scales measured empowerment (10 items), ownership (10), involvement (7), stickiness (10), and evaluation (2). After Harman’s single-factor and CFA tests, structural equation modeling (LISREL 8.8) tested six hypotheses.

**Results:**

All scales showed high reliability (*α* ≥ .82) and convergent validity (AVE > .50, CR > .70). Empowerment positively predicted stickiness (*β* = .73, p < .001) and ownership (*β* = .74, p < .001). Involvement predicted stickiness (*β* = .90, *p* < .001) and ownership (*β* = .95, *p* < .001). Ownership strongly influenced stickiness (*β* = .95, *p* < .001). Stickiness enhanced course evaluation (*β* = .87, p < .001), explaining 76 % of its variance.

**Discussion:**

Results support the empowerment → ownership/involvement → stickiness → evaluation chain, highlighting the need for autonomy-supportive, ownership-fostering pedagogies to sustain engagement and positive course ratings in digital learning environments.

## 1 Introduction

In the field of education, student participation has always been a core factor influencing teaching effectiveness and learning outcomes. In recent years, the increasing adoption of online and blended learning formats in higher education has created new demands on how students engage and persist in digital environments. While such modalities provide greater flexibility, they are also associated with risks of cognitive overload, psychological isolation, and ultimately, disengagement (Holzer et al., 2021). One emerging concern is the difficulty in sustaining students’ continued emotional and behavioral involvement, a phenomenon sometimes referred to as “class stickiness”—the enduring commitment students develop toward a course over time. Student class participation is no longer limited to passive acceptance within a physical space but has gradually evolved into an active, continuous, and multidimensional interactive process. Against this backdrop, how to enhance class stickiness has become an important issue of common concern for educational managers and researchers. In this context, this paper introduces the construct of class stickiness, defined as a student’s sustained psychological presence and emotional commitment to a specific learning environment, which not only directly affects students’ learning outcomes and satisfaction but also relates to the long-term development of education.

Unlike momentary task engagement, stickiness reflects long-term behavioral persistence and affective loyalty, echoing the “customer stickiness” framework from service marketing, where consumers remain committed not only because of satisfaction, but due to perceived autonomy, identity alignment, and emotional connection. In educational settings, understanding what makes students stay—not just show up—has become a pressing concern for both researchers and practitioners.

To explain the emergence and persistence of class stickiness, this study draws on two foundational constructs from motivational and organizational psychology: psychological empowerment and psychological ownership. Psychological empowerment, as originally defined by Spreitzer (1995), encompasses four dimensions—meaning, competence, self-determination, and impact—and serves as a key antecedent of intrinsic motivation across educational and organizational contexts. Recent educational research has extended this model to account for how students’ perceived autonomy, influence, and meaningful participation shape long-term academic outcomes. For instance, Shukla and Arora (2023) demonstrated that when students believe they have the right to choose, be informed, and influence the learning process, they are more likely to exhibit sustained class engagement and commitment.

However, empowerment alone may be insufficient unless students internalize a sense of responsibility and personal ownership over their learning process. This brings into focus psychological ownership—the affective state in which learners perceive a course or learning experience as “mine.” Ownership is triggered when students have autonomy in shaping learning goals and feel accountable for academic outcomes (Gu, 2021). As the “Own it, Learn it, Share it” framework suggests (Lee and Hannafin, 2016), designing opportunities for students to take ownership not only enhances agency, but also deepens their affective commitment and investment in learning. In this regard, empowerment fuels ownership, and ownership translates into class stickiness, manifested through voluntary persistence, emotional loyalty, and the intention to return and recommend.

While the individual roles of empowerment and ownership have been discussed in prior literature, few empirical models have integrated these constructs to explain long-term student engagement. Letzel et al. (2023) highlighted this gap, pointing to the lack of validated frameworks that capture the full motivational sequence from empowerment to sustained commitment. This gap is particularly salient in digital learning environments, where disengagement remains a pervasive challenge. Addressing this need, the current study proposes and empirically tests a structural model that links student psychological empowerment, psychological ownership, and class stickiness, while also examining their collective influence on student class evaluation.

Moreover, the model incorporates student involvement—defined as engagement based on intrinsic interest and personal relevance—as an additional motivational antecedent of both ownership and stickiness. This multidimensional approach reflects the evolving needs of today’s diverse learners, especially in post-pandemic hybrid and online contexts. In these settings, traditional class management strategies are increasingly insufficient, making it essential to design learning environments that foster autonomy, identity, and sustained connection.

By conceptualizing students as “customers” and the class as a “service” environment, this study integrates customer engagement theory with psychological constructs from education to offer a comprehensive model of sustained engagement. Ultimately, this research contributes both theoretically and practically by advancing understanding of how empowerment, ownership, and involvement shape class stickiness and downstream student outcomes. It offers actionable insights for educators and administrators seeking to build inclusive, motivating, and retention-enhancing learning environments.

While the literature on student engagement is extensive, there is a clear gap in understanding how psychological empowerment and ownership interact to produce sustained behavioral and emotional commitment in blended and online class. Most prior studies treat engagement as a short-term or situational response, often overlooking the longitudinal motivational processes that lead to stickiness—the desire to return, re-engage, and recommend. Furthermore, existing models rarely account for the role of identity and learner agency in shaping commitment trajectories.

This study fills these gaps in several ways. First, it operationalizes class stickiness as a distinct construct that incorporates both affective loyalty and behavioral persistence. Second, it integrates psychological empowerment and ownership into a sequential theoretical framework that accounts for their individual and mediating effects on stickiness. Third, it introduces student involvement as a parallel motivational factor that directly contributes to both ownership and stickiness. Finally, the model is empirically tested using data from hybrid learning environments—settings where these dynamics are particularly critical.

Based on the research gaps and contributions identified, this study aims to answer the following key questions: (1) How does student psychological empowerment influence class stickiness directly and indirectly? (2) What roles do psychological ownership and student involvement play in mediating this relationship? (3) Does class stickiness significantly influence students’ class evaluations?

## 2 Literature review and hypothesis development

### 2.1 Class stickiness: the shifting paradigm of engaged learning

The concept of *class stickiness* finds its theoretical roots in the evolving literature on customer engagement in marketing and service psychology. Traditionally, customer engagement is conceptualized as the depth of a consumer’s cognitive, emotional, and behavioral investment in their interactions with a brand or company (Higgins and Scholer, 2009; Hollebeek, 2011; Mollen and Wilson, 2010). It emphasizes reciprocal participation—not only between customer and company, but also among customer communities (Brodie et al., 2011).

In educational contexts, this concept has been adapted to examine students’ sustained psychological and behavioral commitment to learning environments (Pan, 2023). Much like loyal consumers, students may develop enduring connections to “learning brands” such as courses, instructors, or institutions, particularly when actively engaged in co-constructed educational experiences (Gu, 2021; Lee and Hannafin, 2016). This paper reframes engagement through the lens of class stickiness, emphasizing students’ sustained emotional attachment and behavioral intention to remain involved in a specific class context.

In this analogy, students act as empowered participants—or “customers”—within the class, which functions as a branded service space. Especially in hybrid and online learning environments post-COVID, this comparison gains relevance as instructors strive to cultivate not only participation, but also retention and return intent. (Holzer et al., 2021) Martin and Bolliger and Martin (2021) underscore that virtual class demand new forms of engagement, where emotional resonance and meaningful interaction are crucial for fostering connection. As Pan (2023) notes, learners’ sense of autonomy and perceived impact strongly influence their continued participation in digital settings.

Student involvement, a well-established construct in educational psychology, relates to academic performance (Reyes et al., 2012), psychological health, and social development (Steele and Fullagar, 2009). According to Astin (1984), student engagement includes enthusiasm and behavioral participation, as well as emotional commitment. This study adopts Fredricks et al. (2004)‘s tripartite framework—cognitive, emotional, and behavioral engagement—as the operational base for class engagement. Cognitive engagement entails mental investment and sustained attention (Reeve and Tseng, 2011), emotional engagement involves feelings of belonging and connection (Fredricks et al., 2004), and behavioral engagement manifests in participation, collaboration, and academic persistence (Appleton et al., 2008). Lin (2025) further demonstrated that digital co-presence between students and instructors significantly enhances engagement through reciprocal visibility and identity recognition, as learners and teachers co-construct meaning in real-time online interactions.

However, class engagement—often defined as situational, task-specific participation—does not fully capture the enduring loyalty or voluntary return behavior that some students exhibit. To address this gap, this paper introduces the concept of class stickiness—a construct that integrates sustained psychological presence with behavioral loyalty. Distinct from transient engagement, stickiness reflects enduring commitment, akin to brand loyalty in marketing (Watson, 2025).

Whereas engagement may fluctuate with content or instructional style, and attachment reflects stable emotional ties (Libbey, 2004), class stickiness encompasses both dimensions—students’ emotional connection to the class and their voluntary intention to re-engage. Košir et al. (2025) demonstrate that affective attachment to teachers and school significantly lowers dropout risk in hybrid environments, underscoring the value of long-term emotional connection in educational persistence (Table 1).

**Table 1:** Conceptual comparison of engagement, attachment, and stickiness.

Dimension	Engagement	Attachment	Stickiness
Theoretical origin	Educational Psychology (Fredricks et al., 2004)	Attachment and School Bonding Theory (Libbey, 2004; Košir et al., 2025)	Customer Engagement Theory (Van Doorn and Verhoef, 2010)
Core focus	Participation in academic tasks	Emotional connection to school or teachers	Psychological persistence and loyalty to classroom experience
Time orientation	Short-term and task-specific	Mid- to long-term relationship	Long-term, return-based, and loyalty-driven
Indicators	Behavioral, emotional, and cognitive scales	Affective bonding, belonging surveys	Intention to return, recommend, continued involvement
Emotional component	Moderate – linked to academic enthusiasm	Strong emotional ties and trust	Emotional and behavioral commitment to classroom identity
Behavioral expression	Class participation, attention, homework	Less visible – inferred from narratives or surveys	Voluntary re-engagement, classroom identification
Educational role	Process variable – driver of performance	Relational environment – context variable	Outcome variable – reflects sustained value perception
Illustrative example	Raising hand, focused discussion	Feeling secure and supported by teachers	Choosing same instructor or course again; peer recommendation

In this framework, stickiness represents a composite outcome: emerging from engagement, reinforced by attachment, and sustained by student empowerment and ownership. It is marked by both emotional loyalty and intention to return or recommend—a form of educational brand advocacy.

Therefore, this paper thus defines *class stickiness* as the sustained psychological presence and affective loyalty students exhibit toward a learning environment—rooted in psychological empowerment, ownership, and identity formation. It extends beyond transient engagement or passive attachment, emphasizing intentional return and emotional commitment sticky learning spaces—those that foster inclusion, safety, and recognition—are particularly vital for student retention and satisfaction, especially in post-pandemic hybrid class. Understanding why students stay, not just why they show up, becomes essential for long-term educational design.

### 2.2 Student psychological empowerment and class stickiness

Psychological empowerment is defined as a cognitive–motivational state characterized by feelings of meaning, competence, self-determination, and impact (Spreitzer, 1995). These dimensions collectively describe how individuals experience agency and significance in their roles. In education, psychological empowerment captures students’ perceptions of autonomy (having choice), competence (feeling capable), impact (influencing outcomes), and meaningfulness (aligning with personal values), which directly influence their class participation and long-term engagement.

The roots of psychological empowerment can be traced to Self-Determination Theory (Ryan and Deci, 2000), which posits that autonomy, competence, and relatedness are essential for intrinsic motivation and well-being, and to Social Cognitive Theory (Bandura et al., 1997), which highlights the role of self-efficacy in behavior regulation. Together, these frameworks inform how students internalize responsibility for learning and develop persistent engagement behaviors. In a recent structural equation model, Zheng and Xiao (2024) found that self-efficacy—a key component influenced by psychological empowerment—positively predicted both self-regulated learning behaviors and course satisfaction among online learners. Moreover, informal digital learning experiences were also found to enhance this relationship by offering autonomy-supportive environments. Their study reinforces that when students feel psychologically empowered—through confidence and control—they are more likely to invest in sustained learning, leading to deeper class attachment and behavioral loyalty.

Recent empirical work has validated this relationship in contemporary educational settings. For instance, Pan (2023) found that learner empowerment—comprising perceived autonomy, competence, and impact—significantly predicted behavioral engagement in online learning, with autonomy partially mediating this effect. Similarly, Lee et al. (2023) reported that satisfying autonomy and competence needs predicted self-regulated learning and course persistence in online university courses. These studies underscore that empowered students are more likely to invest sustained effort and show loyalty to class environments.

Empowerment also functions indirectly. It fosters a sense of psychological ownership—a student’s internalized belief that “this is my class” or “my learning space.” This ownership, in turn, reinforces behavioral persistence and affective loyalty, both components of class stickiness. As shown by Zhang and Bartol (2010) in workplace contexts and extended to educational settings by Pierce et al. (2001), perceived control and meaningful involvement lead to heightened affective commitment and discretionary effort.

In summary, psychological empowerment builds the internal foundation for student persistence and re-engagement, thereby reinforcing class stickiness.

Therefore, the following hypotheses are proposed:

*H1*: Student psychological empowerment has a direct positive impact on class stickiness.

*H2*: Student psychological empowerment has a direct positive impact on student psychological ownership.

### 2.3 Student psychological ownership and class stickiness

Psychological ownership refers to the state in which individuals feel as though a target of ownership (material or immaterial) is “theirs,” regardless of formal property rights. The theory, originally proposed by Pierce et al. (2001), identifies three core routes through which psychological ownership develops: controlling the target, intimately knowing the target, and investing the self into the target. These psychological routes foster a sense of possession, responsibility, and self-extension (Avey et al., 2009).

In educational contexts, student psychological ownership denotes learners’ perceived ownership over their learning environment or content—such as the feeling that “this class is mine.” It encompasses emotional attachment, identity projection, and a sense of obligation to protect or improve the class community. According to Van Dyne and Pierce (2004), this psychological attachment leads individuals to safeguard and invest more deeply in the object of ownership.

Recent studies validate this mechanism in online and hybrid education. For instance, Holzer et al. (2021) found that when university students felt autonomy, competence, and relatedness (basic psychological needs), they not only self-regulated their learning more effectively but also reported increased identification with digital learning spaces—an antecedent to psychological ownership. Similarly, Pan (2023) showed that learner empowerment predicts persistent engagement through a mediated pathway of intrinsic motivation and sense of responsibility, further supporting the empowerment → ownership → engagement logic.

Furthermore, Zhang and Bartol (2010) highlight that psychological ownership enhances proactive behaviors and personal investment in organizational settings, effects that translate well to educational environments. When students internalize ownership over the class, they are more likely to exhibit class-aligned behaviors such as helping peers, returning voluntarily, and recommending the course—core dimensions of class stickiness.

Therefore, psychological ownership is a critical emotional-cognitive mechanism through which empowerment translates into durable loyalty and re-engagement behavior, the following hypothesis is proposed:

*H3*: Student psychological ownership has a direct positive impact on class stickiness.

### 2.4 Student involvement and class stickiness

Student involvement, distinct from transient attention or behavioral engagement, refers to the psychological relevance that students perceive between themselves and a learning activity, driven by intrinsic needs and personal interests (Zaichkowsky, 1994). According to Self-Determination Theory (Miller et al., 1988), involvement emerges when students’ basic needs—autonomy, competence, and relatedness—are met through emotionally engaging learning environments.

Involvement reflects deep-level processing and voluntary cognitive investment, which contributes to the student’s feeling of being part of a learning community. Wong and Liem (2021) emphasized that involvement represents the motivational energy that sustains academic engagement over time, distinguishing it from surface-level participation.

Post-pandemic studies confirm that students’ emotional and motivational states shape their engagement and learning intentions. Holzer et al. (2021) demonstrated that motivation and achievement emotions were powerful predictors of students’ engagement intensity in hybrid university learning, particularly when learning environments addressed students’ internal values.

In practical learning contexts, involvement fosters the type of emotional and behavioral loyalty that aligns with the class stickiness construct. In emotionally rich, autonomy-supportive learning environments, students report a stronger desire to return, re-engage, and recommend the course. Samson (2025) confirmed this by showing that high-involvement environments in nursing education significantly enhanced both engagement and long-term retention through emotional safety and student-led participation.

Furthermore, involvement may serve as an antecedent to psychological ownership. As students perceive the class to align with their personal interests, they begin to integrate it into their self-concept—producing greater affective loyalty and responsibility. As observed by Holzer et al. (2021), when students’ psychological needs are met, they internalize greater responsibility and sustained behavioral patterns, supporting the link from involvement to ownership and, ultimately, stickiness.

Therefore, the following hypotheses are proposed:

*H4*: Student involvement has a direct positive impact on class stickiness.

*H5*: Student involvement has a direct positive impact on student psychological ownership.

### 2.5 The impact of class stickiness on student class evaluation

Class evaluation is similar to customer loyalty, which is defined as the loyalty and satisfaction that customers develop toward a brand based on continuous participation and involvement (Van Doorn and Verhoef, 2010). In educational settings, this refers to students’ satisfaction with the class experience and their willingness to recommend or re-enroll in similar learning environments. According to customer loyalty theory (Oliver, 1999), customers’ continuous participation and involvement directly affect their loyalty and satisfaction toward a brand. In educational settings, class engagement directly affects students’ overall evaluation and satisfaction with the class by enhancing their continuous participation and involvement (Fredricks et al., 2004). Customer engagement has been shown to significantly enhance customer loyalty (Brodie et al., 2011; Verhoef et al., 2010).

Similarly, in educational settings, class engagement enhances class evaluation by strengthening students’ continuous participation. When students exhibit emotional attachment and continuous engagement, they are more likely to rate the class positively and recommend it to peers. This emotional bonding mirrors consumer loyalty models and has been validated in student retention research (Reeve and Cheon, 2021). Watson (2025) emphasizes that for marginalized students, sticky learning environments—where safety, identity, and connection are established—lead to significantly higher positive evaluations and retention.

Thus, class stickiness not only sustains participation but isignificantly enhances students’ overall satisfaction and their likelihood of recommending the course or re-engaging in future offerings.

Therefore, the following hypothesis is proposed:

*H6*: class stickiness has a direct positive impact on student class evaluation.

**Figure 1:**
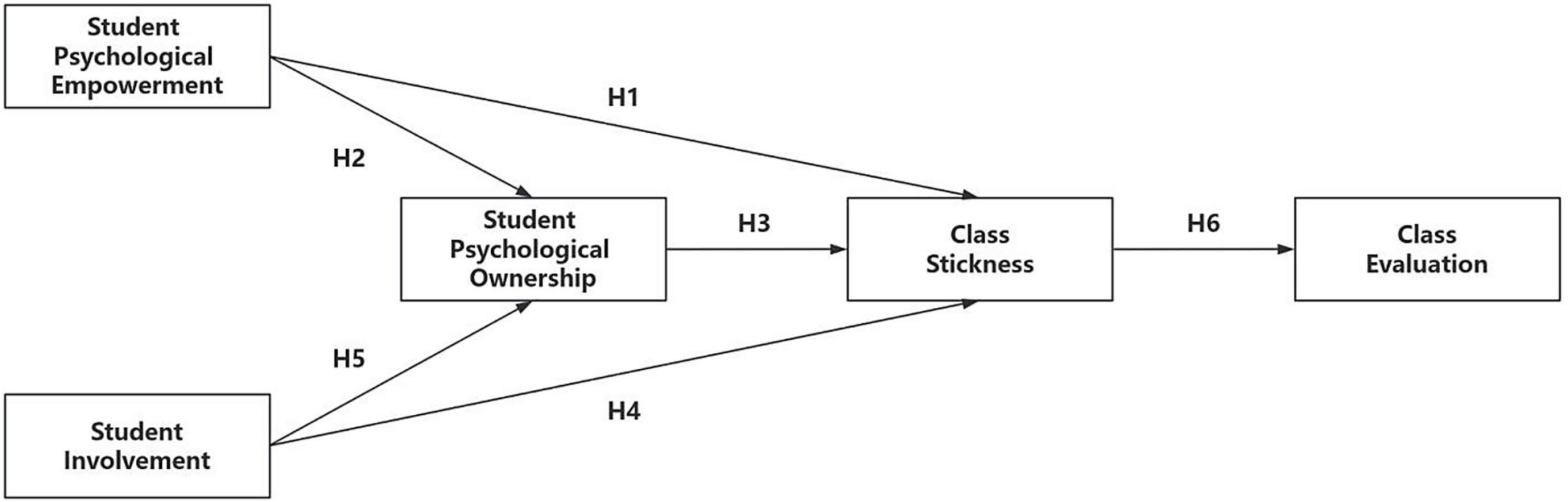
Hypothesis model.

As shown in Figure 1, the hypothesis model is proposed.

## 3 Research design

### 3.1 Variable definition and measurement

#### 3.1.1 Student involvement

This paper defines student engagement as students’ perception of their relevance to the class based on their intrinsic needs and interests. Drawing on Zaichkowsky’s (1994) customer engagement scale, this study measures student engagement from the dimensions of importance, relevance, value, interest, excitement, attractiveness, and necessity.

#### 3.1.2 Student psychological empowerment

This paper employs a well-established scale (Han and Feng, 2012), adapting the context from customers and companies to students and class, and modifying the questions based on the characteristics of teaching scenarios and teacher-student interactions. The scale measures student psychological empowerment from three aspects: the right to choose, the right to be informed, and influence.

#### 3.1.3 Student psychological ownership

The measurement of psychological ownership primarily involves scales and experimental methods. Scales are the most commonly used method, with Van Dyne and Pierce's (2004) psychological ownership scale being one of the most widely used tools. This scale measures psychological ownership from cognitive, emotional, and behavioral dimensions and has high reliability and validity. This paper draws on You et al.’s (2011) modified scale based on Van Dyne and Pierce's (2004) scale, making adjustments according to local characteristics and student traits. The scale consists of three dimensions: “self-concept,” “attitude,” and “sense of responsibility.”

#### 3.1.4 Class stickiness

This paper defines class stickiness as students’ continuous participation, involvement, and interactive behaviors in the class, reflecting their positive attitudes and behavioral manifestations toward the class. Drawing on Vivek et al. (2012) well-established scale, this study measures class stickiness from the dimensions of enthusiasm, conscious participation, and social interaction, modifying the items according to the characteristics of the actual teaching context.

#### 3.1.5 Class evaluation

Student class evaluation, similar to customer loyalty, refers to students’ overall satisfaction and evaluation of the class. Based on Kassim and Asiah Abdullah’s (2010) customer loyalty scale, this study measures class evaluation from the dimensions of attitudinal loyalty and behavioral loyalty. In the specific context of teaching, student evaluation of teaching directly reflects students’ feelings about the course, while recommending the course to other students reflects their true attitudes. Therefore, the scale is designed as Table 2.

**Table 2:** Summary of scales and items.

Variable	Dimension	Item number	Item content
Student involvement		I1	This class is very important to me.
		I2	This class is very interesting.
		I3	This class is highly relevant to me.
		I4	This class is very exciting.
		I5	This class is very meaningful.
		I6	This class is very attractive.
		I7	This class is very necessary.
Student psychological empowerment	Right to choose	E1	I can choose different ways to attend classes.
		E2	I can choose different ways to learn the content.
		E3	I can choose different assignment formats.
	Right to be informed	E4	I can understand the course arrangement from the course website/learning system.
		E5	I can understand the specific content of the course from the course website/learning system.
	Influence	E6	The teacher will adjust the teaching method according to my requests.
		E7	The teacher will add some additional content according to my requests.
		E8	The teacher will try to teach according to my wishes.
		E9	The teacher will design the classroom content according to my specific situation.
		E10	The teacher can provide targeted guidance according to my needs.
Student psychological ownership	Self-concept	O1	I feel that this class belongs to us students.
		O2	I feel a high degree of personal ownership in the course.
		O3	This course has a lot of personal significance to me.
		O4	Most people feel that the course belongs to everyone.
	Attitude	O5	I like this course.
		O6	I am proud to be a student of this course.
		O7	I like the teacher of this course.
		O8	I feel satisfied with this course.
	Sense of responsibility	O9	I always attend classes seriously.
		O10	I complete assignments and other tasks very seriously.
Class stickiness		CS1	I spend a lot of time on this course.	CS2	I am very fascinated by participating in this course.
	CS3	I am very enthusiastic about attending this course.
	CS4	If I do not take this course, my major studies will be affected.
	CS5	Anything related to this course can attract my attention.
	CS6	I want to learn more about this course.
	CS7	I pay close attention to information related to this course.
	CS8	I really enjoy participating in this course with friends.
	CS9	I feel better when I take this course with my friends.
	CS10	I find it more interesting when people around me participate in tasks of this course.
	Classroom evaluation		CE1	I will to give a high score in the teaching evaluation.
CE2			I will to recommend this course to other students.

### 3.2 Questionnaire design

#### 3.2.1 Questionnaire structure

The questionnaire is designed to assess the impact of student psychological empowerment on class stickiness and consists of six main sections, each targeting different research variables for measurement. The first five sections use matrix scale questions, scored on a seven-point Likert scale ranging from 1 to 7, representing “strongly disagree” to “strongly agree.” The sixth section collects demographic information using single-choice and fill-in-the-blank questions.

##### 3.2.1.1 Student involvement

Drawing on Zaichkowsky’s (1994) customer engagement scale, this section measures student engagement from the dimensions of importance, interest, relevance, excitement, meaningfulness, attractiveness, and necessity.

##### 3.2.1.2 Student psychological empowerment

Based on Han and Feng (2012) scale, this section measures student psychological empowerment from the dimensions of the right to choose, the right to be informed, and influence.

##### 3.2.1.3 Student psychological ownership

This section uses You et al.’s (2011) modified scale based on Van Dyne and Pierce’s (2004) scale, measuring student psychological ownership from the dimensions of self-concept, attitude, and sense of responsibility.

##### 3.2.1.4 Class stickiness

Drawing on Vivek et al. (2012) customer engagement scale, this section measures class stickiness from the dimensions of enthusiasm, conscious participation, and social interaction.

##### 3.2.1.5 Class evaluation

This section uses self-designed questions to assess students’ overall evaluation of the course.

##### 3.2.1.6 Demographic information

This section collects basic information such as gender, age, major, and course name, which will be used for subsequent stratified analysis and result interpretation.

#### 3.2.2 Pilot testing

Before the formal distribution of the questionnaire, a pilot test was conducted to assess the understandability and reliability of the questionnaire. The pilot test was carried out on a small sample of students, and the results showed that the questionnaire had good understandability and acceptability, with Cronbach’s *α* coefficients all above 0.8, indicating good internal consistency of the scales.

### 3.3 Data source and collection

The questionnaire was published online on the Credamo platform to ensure the convenience and wide coverage of data collection. At the same time, the respondents were limited to students with a maximum education level of undergraduate, ensuring that the questionnaire link was shared with the target student population. During the questionnaire completion process, anonymity and voluntariness were ensured to improve the authenticity and validity of the data. A total of 320 valid questionnaires were collected for subsequent data analysis.

## 4 Data analysis

### 4.1 Common method bias test

The existence of common method bias may lead to misjudgment of the relationships between variables, thereby affecting the accuracy of research conclusions. Therefore, this paper conducted Harman single-factor tests and confirmatory factor analysis tests on all measured variables to identify whether common method bias existed.

As shown in Tables 3, 4, the variance explained by the first factor before rotation was 44.545%, less than 50%, indicating that there was no serious common method bias (Hair, 1998).

**Table 3:** Variance explained.

Factor number	Eigenvalue			Variance explained before rotation			Variance explained after rotation		
	Eigenvalue	Variance explained before rotation	Cumulative variance explained before rotation	Eigenvalue	Variance explained before rotation	Cumulative variance explained before rotation	Eigenvalue	Variance explained before rotation	Cumulative variance explained after rotation
1	17.373	44.545	44.545	17.373	44.545	44.545	11.985	30.732	30.732
2	2.848	7.303	51.848	2.848	7.303	51.848	6.402	16.416	47.148
3	2.116	5.426	57.274	2.116	5.426	57.274	3.949	10.126	57.274

**Table 4:** Confirmatory factor analysis (CFA) results.

Common Indicators	*χ* ^2^	df	*p*	*χ*^2^/df	GFI	RMSEA	RMR	CFI	NFI	NNFI
Judgment criteria	–	–	>0.05	<3	>0.9	<0.10	<0.05	>0.9	>0.9	>0.9
Values	3343.524	702	0.000	4.763	0.571	0.109	0.173	0.723	0.675	0.708
Other indicators	TLI	AGFI	IFI	PGFI	PNFI	PCFI	SRMR	RMSEA 90% CI		
Judgment criteria	>0.9	>0.9	>0.9	>0.5	>0.5	>0.5	<0.1	–		
Values	0.708	0.524	0.724	0.514	0.639	0.685	0.085	0.097 ~ 0.114		

For the Default Model, *χ*^2^(741) = 10285.524, *p* = 1.000. AIC = 135.103, BIC = 429.032.

### 4.2 Reliability and validity analysis

#### 4.2.1 Reliability analysis

Reliability analysis was conducted on each scale. The Cronbach’s α coefficients for all scales were above 0.8, indicating good internal consistency. Specifically, the Cronbach’s α for the student engagement scale was 0.818, for the student psychological empowerment scale was 0.877, for the student psychological ownership scale was 0.907, for the class stickiness scale was 0.883, and for the class evaluation scale was 0.839. The results show that the designed scales have high reliability in this study.

#### 4.2.2 Validity analysis

The applicability of the scales was assessed through KMO and Bartlett tests. As shown in Table 5, the results showed that the KMO values for all variables were greater than 0.8, and the *p*-values for Bartlett’s test of sphericity were all less than 0.001, indicating that the data were suitable for factor analysis.

**Table 5:** KMO and bartlett test results.

Variable	KMO value	Bartlett’s Test of Sphericity		
		Approx. Chi-Square	df	*p*
Student psychological empowerment	0.856	1685.396	45	<0.001
Student involvement	0.805	1189.797	21	<0.001
Student psychological ownership	0.917	1963.174	45	<0.001
Class stickiness	0.888	2212.310	45	<0.001
Class evaluation	0.500	244.734	1	<0.001

Additionally, confirmatory factor analysis was performed using the maximum likelihood estimation procedure in LISREL software, with the covariance matrix as the input matrix. The analysis results showed that the normed fit index (NFI) was 0.75, the non-normed fit index (NNFI) was 0.78, the comparative fit index (CFI) was 0.80, the incremental fit index (IFI) was 0.80, the goodness of fit index (GFI) was 0.64, the adjusted goodness of fit index (AGFI) was 0.60, the relative fit index (RFI) was 0.73, indicating high discriminant validity of the data.

### 4.3 Second-order factor analysis

To further verify the structural validity of the student psychological empowerment and student psychological ownership scales, this study continued with second-order factor analysis, analyzing the three dimensions of student psychological empowerment (right to choose, right to be informed, and influence) and the three dimensions of student psychological ownership (self-concept, sense of responsibility, and attitude).

#### 4.3.1 Student psychological empowerment

The student psychological empowerment scale includes three first-order factors: the right to choose (E1-E3), the right to be informed (E4-E5), and influence (E6-E10). As shown in Tables 6, 7, the AVE values for these first-order factors were 0.533, 0.630, and 0.680, respectively, and the CR values were 0.772, 0.773, and 0.914, all meeting the requirements for convergent validity (AVE > 0.5, CR > 0.7), indicating good internal consistency of the scale. The second-order factor analysis results showed that the first-order factor structure of student psychological empowerment had a stable higher-order structure. The model fit indices were *χ*^2^/df = 2.856, GFI = 0.947, RMSEA = 0.076, RMR = 0.093, and CFI, NFI, and NNFI were all close to or exceeded 0.95, indicating a good fit between the second-order factor model and the data.

**Table 6:** AVE and CR indicators for student psychological empowerment model.

Factor	Average variance extracted (AVE) value	Composite reliability (CR) value
Right to choose	0.533	0.772
Right to be informed	0.630	0.773
Influence	0.680	0.914

**Table 7:** Model fit indices for student psychological empowerment model.

Common Indicators	*χ* ^2^	df	*p*	*χ*^2^/df	GFI	RMSEA	RMR	CFI	NFI	NNFI
Judgment criteria	–	–	>0.05	<3	>0.9	<0.10	<0.05	>0.9	>0.9	>0.9
Values	91.377	32	0.000	2.856	0.947	0.076	0.093	0.964	0.947	0.950
Other indicators	TLI	AGFI	IFI	PGFI	PNFI	PCFI	SRMR	RMSEA 90% CI		
Judgment criteria	>0.9	>0.9	>0.9	>0.5	>0.5	>0.5	<0.1	-		
Values	0.950	0.909	0.965	0.551	0.673	0.686	0.036	0.058 ~ 0.095		

For the Default Model, *χ*^2^(45) = 1713.054, *p* = 1.000. AIC = 45.429, BIC = 132.100.

#### 4.3.2 Student psychological ownership

The student psychological ownership scale includes three first-order factors: self-concept (O1-O4), sense of responsibility (O9-O10), and attitude (O5-O8). As shown in Tables 8, 9, the AVE values for these first-order factors were 0.515, 0.744, and 0.585, respectively, and the CR values were 0.803, 0.897, and 0.736, all meeting the requirements for convergent validity, indicating good internal consistency of the scale. The second-order factor analysis results showed that the first-order factor structure of student psychological ownership had a stable higher-order structure. The model fit indices were *χ*^2^/df = 2.279, GFI = 0.963, RMSEA = 0.063, RMR = 0.051, and CFI, NFI, and NNFI were all close to or exceeded 0.97, indicating a good fit between the second-order factor model and the data.

**Table 8:** AVE and CR indicators for student psychological ownership model.

Factor	Average variance extracted (AVE) value	Composite reliability (CR) value
Self-concept	0.515	0.803
Sense of responsibility	0.744	0.897
Attitude	0.585	0.736

**Table 9:** Model fit indices for student psychological ownership model.

Common Indicators	*χ* ^2^	df	*p*	*χ*^2^/df	GFI	RMSEA	RMR	CFI	NFI	NNFI
Judgment criteria	–	–	>0.05	<3	>0.9	<0.10	<0.05	>0.9	>0.9	>0.9
Values	54.700	24	0.000	2.279	0.963	0.063	0.051	0.980	0.965	0.970
Other indicators	TLI	AGFI	IFI	PGFI	PNFI	PCFI	SRMR	RMSEA 90% CI		
Judgment criteria	>0.9	>0.9	>0.9	>0.5	>0.5	>0.5	<0.1	-		
Values	0.970	0.931	0.980	0.514	0.643	0.653	0.029	0.041 ~ 0.086		

For the Default Model, *χ*^2^(36) = 1572.651, *p* = 1.000. AIC = 41.658, BIC = 120.793.

In summary, the second-order factor analysis provided additional evidence supporting the application of latent variables such as student psychological empowerment and student psychological ownership in the field of education. Future research can further explore the stability and applicability of these higher-order structures in different educational environments and cultural backgrounds.

### 4.4 Structural equation modeling

As shown in Table 10, the model estimation results showed that the path coefficient from student psychological empowerment to class stickness was 0.73, with a t-value of 24.00 and a significance level of *p* < 0.001. This indicates a significant positive relationship between student psychological empowerment and class stickness, supporting Hypothesis H1. The path coefficient from student psychological empowerment to student psychological ownership was 0.74, with a t-value of 25.15 and a significance level of *p* < 0.001. The results show that student psychological empowerment significantly positively influences student psychological ownership, supporting Hypothesis H2. The path coefficient from student psychological ownership to class stickness was 0.95, with a t-value of 99.45 and a significance level of *p* < 0.001. This indicates that student psychological ownership significantly positively influences class stickness, supporting Hypothesis H3. The path coefficient from student involvement to class stickness was 0.90, with a t-value of 58.44 and a significance level of *p* < 0.001. The results support Hypothesis H4, indicating that student involvement significantly positively influences class stickness. The path coefficient from student involvement to student psychological ownership was 0.95, with a t-value of 85.15 and a significance level of *p* < 0.001. This indicates that student involvement significantly positively influences student psychological ownership, supporting Hypothesis H5. The path coefficient from class stickness to class evaluation was 0.87, with a *t*-value of 39.97 and a significance level of *p* < 0.001. The results support Hypothesis H6, indicating that class stickness significantly positively influences student class evaluation.

**Table 10:** Standardized estimates for structural equation model.

Relationship between variables	Standardized estimate/*t*-value
Student Psychological Empowerment → Student Psychological Ownership	0.74/25.15
Student Engagement → Student Psychological Ownership	0.95/85.15
Student Engagement → Class Stickiness	0.90/58.44
Student Psychological Empowerment → Class Stickiness	0.73/24.00
Student Psychological Ownership → Class Stickiness	0.95/99.45
Class Stickiness → Class Evaluation	0.87/39.97

## 5 Summary and discussion

### 5.1 Conclusion

#### 5.1.1 Applicability of student psychological empowerment and ownership scales

This study validated the structural applicability of Han and Feng (2012) and You et al.’s (2011) scales in educational contexts. Specifically, student psychological empowerment was reliably represented by the second-order dimensions of choice, informed decision-making, and influence, while psychological ownership consisted of self-concept, responsibility, and attitude.

This finding offers empirical evidence for the transferability of motivation-related scales originally developed in organizational and marketing domains to educational settings. However, it also raises important theoretical questions regarding the contextual specificity of these constructs. For instance, influence in a corporate team may imply decision-making power, while in classrooms it may be limited to feedback participation or low-stakes choices.

Thus, although the scales function psychometrically in the classroom, future research should further refine the operational meaning of these constructs across educational levels and learning cultures.

#### 5.1.2 Impact of student psychological empowerment on class stickiness

The structural equation model confirmed that student psychological empowerment, psychological ownership, and student involvement significantly and positively affect class stickiness. In turn, class stickiness substantially predicted students’ evaluation of the class. These relationships are aligned with the motivational chain proposed in customer engagement theory (Brodie et al., 2011), where engagement is sustained through a sense of autonomy, ownership, and emotional value.

However, this study moves beyond mere correlation by articulating a sequential mechanism: empowerment → ownership → stickiness → evaluation. This framework offers a more granular understanding of how psychological conditions in the classroom transform into durable behavioral loyalty.

While the findings validate earlier propositions about empowerment’s role in engagement (Pan, 2023), the inclusion of ownership as a mediating construct adds novel explanatory power. Ownership transforms short-term motivation into long-term identification, which has often been overlooked in past models that treat engagement as an isolated outcome.

### 5.2 Pedagogical implications

#### 5.2.1 Elevating empowerment beyond surface-level autonomy

While prior literature recommends offering students choice, our findings suggest that empowerment must be substantive—not merely procedural. Simply allowing content selection is insufficient if students do not perceive real influence over learning outcomes or evaluation mechanisms.

In educational settings, student psychological empowerment directly affects their class participation and engagement by strengthening their sense of control and autonomy over the learning process. Therefore, educators should pay attention to the role of student psychological empowerment and enhance students’ sense of control and autonomy by granting them more rights to choose, be informed, and exert influence. Specifically, educators can encourage students to autonomously select learning content and methods through flexible instructional design and diverse learning tasks. For example, educators can provide multiple learning paths and task options, allowing students to choose learning methods according to their interests and abilities. Additionally, teachers can enhance students’ class stickiness by providing transparent course arrangements and timely feedback, thereby strengthening students’ sense of control over the learning process.

#### 5.2.2 Psychological ownership as a process, not a trait

Student psychological ownership is another important factor influencing class stickiness. Psychological ownership theory suggests that when individuals feel a sense of belonging to and ownership of an object, they exhibit a higher desire to protect and engage with it. In educational settings, student psychological ownership enhances students’ continuous participation and engagement in the class by strengthening their sense of identification with and responsibility for the class. Psychological ownership should not be treated as a static disposition but as an evolving relationship between student and learning environment. While collaborative learning and project-based design are helpful, these approaches risk being tokenistic if not paired with ongoing reflection and accountability.

For educators, it is important to focus on cultivating students’ psychological ownership and enhancing their sense of belonging to and responsibility for the class to improve class participation. For example, teachers can enhance students’ sense of belonging to the class through group cooperative learning and project-based learning. In group cooperative learning, students can enhance their sense of belonging to the class by jointly completing tasks; in project-based learning, students can enhance their sense of responsibility for the class by designing and implementing projects on their own. Additionally, teachers can further enhance students’ psychological ownership of the class by encouraging students to participate in class decision-making and course design.

#### 5.2.3 Stimulating students’ intrinsic motivation to enhance class stickness

Student involvement directly affects their continuous participation and engagement in the class by enhancing their interest in and relevance to the class. Educators should pay attention to students’ intrinsic motivation and interests and stimulate their intrinsic needs to enhance class stickness. For example, teachers can design interesting and meaningful learning tasks to stimulate and enhance students’ interest in and engagement with the class. Additionally, teachers can enhance students’ class stickiness by using diverse teaching methods and interactive forms, flexible assignment formats, timely feedback, and personalized guidance. After students continuously participate in and engage with the class, their evaluation of the class will naturally improve.

Although involvement significantly influenced stickiness, its impact was partially mediated by empowerment and ownership. This suggests that mere participation does not guarantee persistence unless it is psychologically anchored. Instructors should therefore distinguish between compelled engagement (e.g., attendance, grade-motivated responses) and volitional engagement arising from personal relevance and agency. Pedagogical strategies should target this deeper level of motivation through real-world tasks and dialogic teaching.

### 5.3 Limitations and future directions

Despite the meaningful findings of this study, there are still some limitations, and future research can further expand on this basis.

First, the data of this study were derived from a cross-sectional survey at a single time point. Future research can adopt a longitudinal research design to track participants over time and collect data at multiple time points to further explore the relationships.

Second, the sample of this study mainly consisted of Chinese university students, and the generalizability of the research findings may be influenced by cultural background and educational systems. Future research can conduct cross-cultural comparisons in different cultural backgrounds and educational systems to verify the generalizability of the research findings. Additionally, the study did not explicitly consider how collectivist values, such as those influenced by Confucian heritage, may shape students’ perceptions of empowerment and belonging. Future studies may explore how cultural values mediate or moderate psychological constructs in different educational settings.

Third, this study primarily focused on the impact of student psychological empowerment, student psychological ownership, and student involvement on class stickiness. Variables such as teacher autonomy support, institutional trust, or technology satisfaction may moderate the empowerment-stickiness pathway. Future research can further explore other potential moderating and mediating variables, such as teachers’ teaching styles and class atmosphere, to gain a more comprehensive understanding of the formation mechanisms of class stickiness.

Fourth, the current model was developed and validated among typically developing university students. However, its applicability to students with learning differences—such as those with cognitive disabilities, non-verbal learning styles, or special education needs—remains unexplored. Future research could investigate how psychological empowerment functions in inclusive education contexts, and whether the constructs of class ownership and engagement manifest differently.

Fifth, while this study was grounded in empowerment theory and customer engagement, future work could integrate perspectives from self-determination theory (SDT) and motivational interviewing (MI) to explore deeper mechanisms underlying behavioral change. Concepts such as empathy, ambivalence, discrepancy, and self-efficacy could enrich our understanding of how students navigate motivation and class relationships.

Sixth, this study employed a quantitative approach to test theoretical relationships. However, qualitative methods such as in-depth interviews, focus groups, or student reflective journals may uncover rich narratives about how students perceive empowerment, ownership, and engagement. Future studies adopting qualitative or mixed-methods designs can provide deeper insights into students’ lived experiences and the contextual factors influencing class stickiness.

These extensions will not only deepen the theoretical foundation of class stickiness but also increase the inclusiveness, applicability, and cross-cultural relevance of future research.

## Data Availability

The datasets presented in this article are not readily available because the dataset is restricted due to privacy concerns and cannot be shared without prior consent from the participants. Requests to access the datasets should be directed to Junhui Zhang, Polaris428@163.com.
